# Impact of Adipose Tissue and Lipids on Skeletal Muscle in Sarcopenia

**DOI:** 10.1002/jcsm.70000

**Published:** 2025-07-10

**Authors:** Soo Yeon Jang, Kyung Mook Choi

**Affiliations:** ^1^ Division of Endocrinology and Metabolism, Department of Internal Medicine Korea University College of Medicine Seoul South Korea

**Keywords:** adipose tissue, cachexia, fat mass, lipids, sarcopenia

## Abstract

**Background:**

Although the decline in muscle mass, function and increased visceral obesity are attracting substantial attention in the ageing society, approved treatment modalities for sarcopenia/sarcopenic obesity (SO) remain limited. Elucidating effects and mechanisms of adipose tissue and lipids on skeletal muscle is important for identifying potential prevention and treatment targets for sarcopenia/SO.

**Methods:**

In this narrative review, we aim to comprehensively summarize current knowledge on how adipose tissue and lipid metabolites influence skeletal muscle with detailed mechanistic explanations, especially in sarcopenia development. We also tried to explore future perspectives for optimal strategies for managing sarcopenia.

**Results:**

Fatty infiltration in skeletal muscle can alter the structure, metabolism and signalling pathways of muscle, thereby worsening muscle function and physical performance. Intracellular lipid droplets could disrupt normal physiology within muscle cells, but it might be influenced not only by quantity but also by size, location and characteristics of lipid droplets. Intracellular lipid metabolites may induce lipotoxicity in cell signalling of muscle cells, but effects might differ by types or chemical structure. Highly trained athletes exhibit insulin sensitivity despite high levels of muscular fat, a phenomenon called the athlete's paradox. Lipid droplets within the skeletal muscle of athletes are small and are mainly located in the intermyofibrillar area, which is rich in fast‐twitch, Type I fibres. In contrast, patients with Type 2 diabetes/obesity accumulate larger lipid droplets in the subsarcolemmal area, which is richer in Type II fibres. Ageing is intricately associated with mitochondrial dysfunction and the concomitant decline in mitochondrial biogenesis, all of which may lead to sarcopenia. SIRT1 and AMPK, two key energy sensors, are involved in mitochondrial biogenesis through regulation of PGC‐1*α*. Modulation of PGC‐1*α* levels in skeletal muscle may help protect against sarcopenia by preserving muscle integrity, enhancing muscle function, improving insulin sensitivity and reducing inflammation and oxidative stress. Excessive nutrient intake and obesity triggers mitochondrial dysfunction by inducing activation of the inflammatory response and increased production of reactive oxygen species. Skeletal muscle and adipose tissue are closely connected through mediators called adipokines and myokines, and it is important to understand the mechanisms of their interaction.

**Conclusions:**

Dysregulation of lipid metabolism and intramuscular fat accumulation leads to inflammation, oxidative stress, insulin resistance and mitochondrial dysfunction, resulting in reduced muscle mass and strength. Further research on associations between fat/lipids and muscle would be helpful to investigate optimal management strategies for sarcopenia/SO in the rapidly ageing world.

## 1 Introduction

With ageing, the human body undergoes changes in its composition. A decline in muscle mass and a gain in fat mass occur, and fat is redistributed, especially to the abdomen, thus exerting metabolically unfavourable effects [1]. Reduced metabolic rates, unhealthy nutrition, decreased physical activity and hormonal changes accompanying ageing partially explain this age‐related body composition shift; however, the exact mechanism has not yet been elucidated [2].

Sarcopenia refers to decreased muscle mass and strength primarily owing to ageing and is associated with an increased risk of falls, frailty, cardiometabolic disorders, neurodegenerative diseases, hospitalization and even mortality [3]. Sarcopenic obesity (SO) is a condition wherein sarcopenia and obesity coexist. Approximately 11% of the old adult population worldwide is estimated to have SO [4], which is projected to increase in the rapidly ageing world. Further, SO exhibits more serious health outcomes than sarcopenia or obesity alone [5], which underscores the synergistic effects of a decrease in muscle mass/function and an increase in adiposity.

An important function of adipose tissue is storing energy in the form of triglycerides to maintain energy homeostasis [6]. In response to overnutrition and physical inactivity (low energy consumption), adipocytes undergo hyperplasia and hypertrophy [7]. When adipose tissue expands excessively, stress and inflammatory signalling pathways are activated owing to stimuli such as insufficient blood supply and hypoxia [7, 8]. Amplified hypoxia‐inducible factor 1*α* promotes angiogenesis, contributing to local and systemic inflammation [9]. Augmented oxidative stress, dysfunctional mitochondria and increased hypoxia are characteristics of defective adipose tissue [10]. Dysfunctional adipocytes exhibit higher lipolytic activity and reduced capacity to uptake free fatty acids, diverting lipids to non‐adipose tissues [8]. Ectopic fat accumulation disturbs cell metabolism in metabolic organs, including the skeletal muscle, liver and heart [11]. Along with its function as an energy reservoir, adipose tissue is an important endocrine organ that secretes diverse signalling molecules called adipokines. Adipokines act in autocrine, paracrine and endocrine manners and mediate communication between fat cells and other organs in the body [12].

In this review, we discuss the various effects of adipose tissue and lipid metabolites on skeletal muscle with detailed molecular mechanisms, particularly emphasizing their impact on pathophysiology of sarcopenia. Despite the increasing burden of disease, pharmacological treatments for sarcopenia remain limited. A deeper understanding of the molecular mechanisms by which fat and lipids affect skeletal muscle will provide an important foundation for identifying novel therapeutic targets for sarcopenia. We also review clinical trials evaluating the impact of obesity or lipid management on sarcopenia and aim to provide a future perspective for developing optimal management strategies for sarcopenia.

## 2 Effects of Fat Accumulation on Skeletal Muscle

**FIGURE 1:**
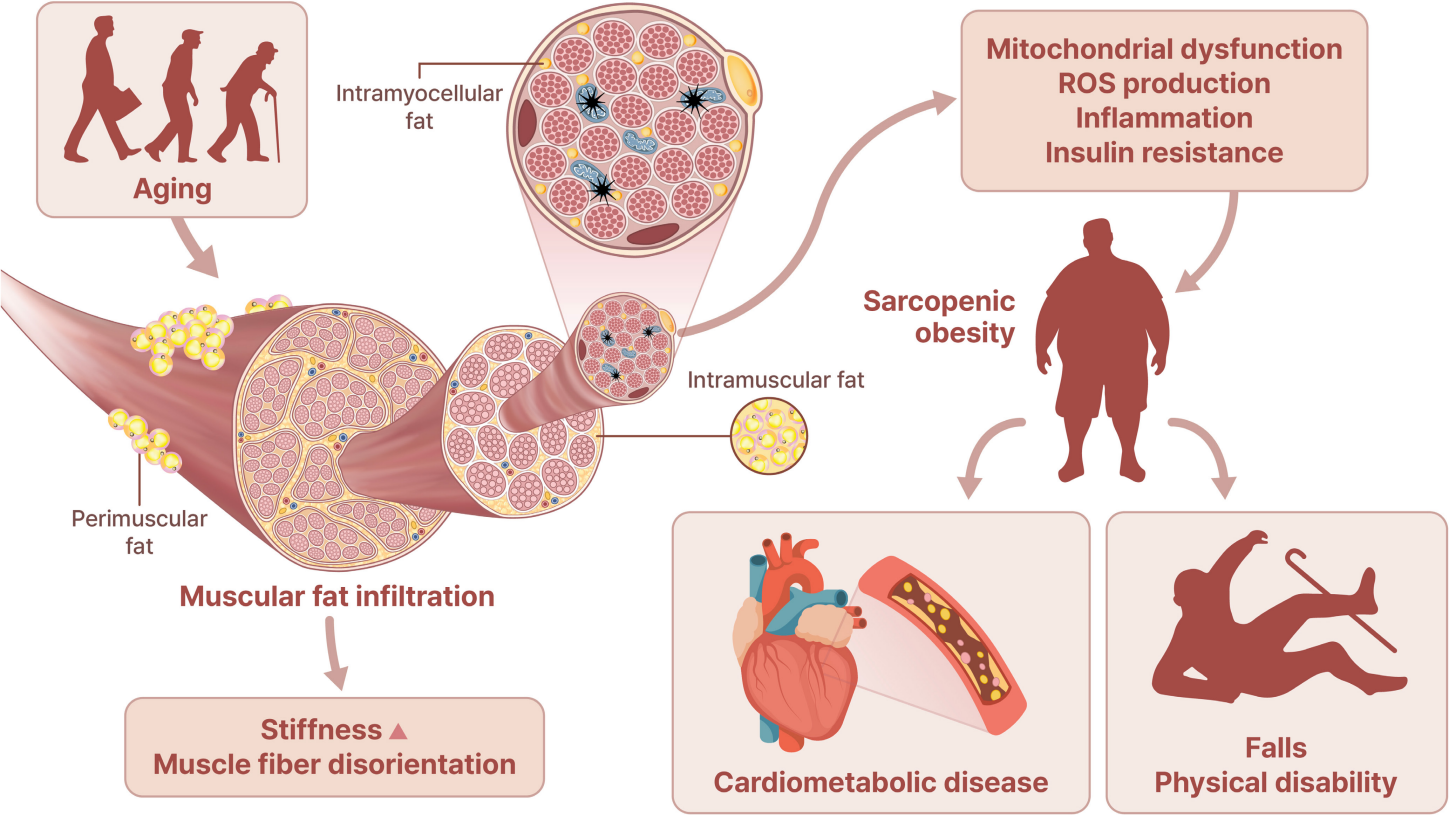
Illustrations on muscular fatty infiltration and their implications.

Fat infiltration in skeletal muscle occurs through two distinct forms: between muscle fibres and within muscle fibres (Figure 1). Intermuscular adipose tissue (IMAT) refers to fat accumulation in the spaces between and surrounding skeletal muscle groups [13]. The origins of cells comprising IMAT are heterogeneous: muscle satellite cells, muscle‐resident fibro‐adipogenic progenitors (FAPs) or mesenchymal progenitors and adipose stromal cells from subcutaneous fat [14]. Adipogenesis occurs under cues associated with contributing factors such as ageing, muscle loss, muscle damage and regeneration [15]. Zinc‐finger protein Zfp423 is known to be a transcriptional regulator that determines the fate of progenitors as adipocytes, and peroxisome proliferator‐activated receptor *γ* (PPAR*γ*) and CCAAT/enhancer‐binding proteins (C/EBPs) expressions are important in the process of differentiation into adipocytes [16]. Complicated transcriptional and signalling pathways are involved in IMAT formation and regulation; however, the exact mechanisms in vivo are yet to be clarified. Intramuscular fat is a fat deposition in the endomysium and perimysium. FAPs are known to be cellular sources of intramuscular fat in conditions such as obesity or ageing and support the activation and differentiation of muscle stem cells (MuSCs) as regulators of skeletal muscle regeneration [17].

Previous studies have demonstrated that muscular fatty infiltration correlates with decreased muscle contractility, which lowers muscle strength [18]. Along with non‐contractile fat tissue replacing the muscle fibres, owing to the proximity to muscle, IMAT or intramuscular fat may affect muscle strength and function by precipitating structural changes such as muscle fibre orientation, pennation angle and fascicle length [19]. Additionally, IMAT around the vessels that feed the muscles impedes the oxygen and nutrient supply through the circulatory system, resulting in a decline in muscle function [20]. In this vein, increased IMAT has been observed among patients with restricted movement or muscle wasting, such as among those after spinal cord injury, thus supporting the significant association between IMAT and decreased muscle function [21]. Rahemi et al. reported that intramuscular fat, with stiffer properties than muscle, can elevate the stiffness of connective tissue, thereby reducing muscle strength and quality in human gastrocnemius muscle [22]. Therkelsen et al. reported increased intramuscular fat, represented by lower muscle attenuation in computed tomography, is associated with slow walking speed in individuals from the Framingham Heart Study [23]. This study used data from paraspinal muscles that do not play a significant role in determining walking speed, and it was difficult to verify the causal relationship between intramuscular fat and physical performance due to the nature of the cross‐sectional study.

Along with structural changes in the muscle, fatty infiltration can influence muscle metabolism, primarily through insulin resistance (Figure 1). Previous studies have indicated that higher IMAT content is observed among insulin‐resistant individuals. Komiya et al. demonstrated that a higher intramuscular fat volume was related to insulin resistance, as indicated by the elevated homeostasis model insulin resistance index (HOMA‐IR) in obese men, although HOMA‐IR may not accurately reflect insulin resistance [24]. Sachs et al. revealed that IMAT reduces insulin sensitivity in human skeletal muscle in vitro and suggested that IMAT may contribute to insulin resistance to the similar extent as visceral adipose tissue [25]. The local concentration of fatty acids would rise as IMAT's lipolytic activity increases compared to subcutaneous adipose tissues, which may contribute to the development of insulin resistance in skeletal muscle [13]. Additionally, ectopic fat deposition in the muscle secretes pro‐inflammatory cytokines and induces immune cell infiltration and polarization into pro‐inflammatory phenotypes, and this inflammatory environment may cause insulin resistance [26]. Local insulin resistance in muscle can precipitate systemic insulin resistance because skeletal muscle is responsible for approximately 70%–80% of insulin‐dependent glucose uptake [27]. Furthermore, insulin acts as an anabolic hormone, and an impaired ability to utilize glucose interferes with normal protein synthesis in muscle, resulting in the loss of muscle mass [28]. In the Korea National Health and Nutrition Examination Survey (KNHANES), the triglyceride‐glucose (TyG) index, a novel surrogate marker of insulin resistance, was positively associated with the risk of low muscle mass in adults aged ≥ 40 years [29]. In the Korean Sarcopenic Obesity Study (KSOS), we reported that Type 2 diabetes was significantly associated with a higher risk of sarcopenia [30] and when diabetes and sarcopenia coexisted, the all‐cause and cardiovascular mortality rates also increased in a longitudinal nationwide study [31]. These studies are based on data from Korean adults; therefore, the results cannot be generalized to other ethnicities. SO would be caused by lipotoxicity and subclinical inflammation in the skeletal muscles and adipose tissue, thus increasing the risk of cardiometabolic diseases [32].

We have discussed that fat deposition in skeletal muscle may play an important role in the pathogenesis of sarcopenia. Since cardiac muscle is also closely related to and affected by lipid metabolism, it would be meaningful to address the effects of fatty infiltration in cardiac muscle as well. Cardiac muscle is more dependent on fatty acids for energy production than skeletal muscle. Excessive lipid supply or mitochondrial dysfunction of the myocardium can cause fatty accumulation and changes in the structure and function of cardiac muscle. Thanassoulis et al. reported that pericardial fat is associated with atrial fibrillation in community‐dwelling middle‐aged and older adults from the Framingham Heart Study cohort [S1]. Kenchaiah et al. showed the linear increase in the risk of heart failure according to pericardial fat volume in those from the Multi‐Ethnic Study of Atherosclerosis (MESA) [S2]. In an observational study of patients with Type 2 diabetes, increased myocardial fat accumulation was related to systolic dysfunction of the left ventricle [S3]. These findings may be due to oxidative stress, proinflammatory responses and consequent fibrosis of cardiac muscle, but discriminative measurement of myocardial/epicardial/pericardial fat is challenging and the specific underlying mechanism has not been fully discovered.

Muscular fatty infiltration could induce changes in the structure and metabolism of skeletal muscle. However, it is not fully clarified how specific changes in muscular configurations, possibly influenced by the quantity or location of fat, affect indices measuring muscle strength or function. Furthermore, it remains to be elucidated whether ectopic fat in muscle acts differently in primary or disease‐related secondary sarcopenia depending on skeletal muscle type.

## 3 Intracellular Fat and Lipids in Skeletal Muscle

Circulatory fatty acids can enter the muscle through passive diffusion and transport via proteins such as cluster of differentiation 36 (CD36) and fatty acid transport proteins (FATP) [33]. Entered fatty acids are either oxidized in the mitochondria, producing energy or stored as lipid droplets within cells. The expression and activity of lipases and their associated proteins regulate the turnover of triacylglycerol and the production of lipid derivatives [34]. Decreased fatty acid oxidation capacity in skeletal muscle has been observed among patients with insulin resistance, Type 2 diabetes and accumulation of intramuscular triglyceride (IMTG) [35].

Metabolic inflexibility, the inability to switch fuel oxidation in response to changes in nutrient availability, results in the accumulation of intramyocellular lipid (IMCL) and is implicated in obesity, sarcopenia and Type 2 diabetes [36]. IMCL refers to lipid droplets that accumulate in the cytoplasm of muscle cells. Prior studies have reported an inverse relationship between IMCL and insulin sensitivity though some differences exist depending on the characteristics of the study participants or muscle types used for the analysis [37]. However, Goodpaster et al. demonstrated that the skeletal muscle of athletes trained for endurance exhibited insulin sensitivity despite high lipid content levels within muscle fibre possibly to satisfy energy requirements [38]. This paradoxical relationship, called the athlete's paradox, suggests that the accumulation of IMCL alone is insufficient to explain insulin resistance.

Lipid droplets in the skeletal muscle of trained athletes are small‐sized with a large surface area, which enables a greater likelihood of contact with mitochondria, primarily located in the intermyofibrillar areas and rich in Type 1 fibres [39]. By contrast, a greater number of large‐sized lipid droplets in the subsarcolemmal region of Type 2 fibres are characteristic of patients with Type 2 diabetes [40]. Moreover, older adults exhibit larger lipid droplets, fewer mitochondria and a smaller proportion of lipid droplets in close connection with mitochondria [40]. The consequences of IMCL on cell metabolism within skeletal muscle fibre differ depending on the distribution, location and characteristics of the lipid droplets, all of which may influence hydrolytic activities and lipid metabolite production.

Exercise as well as high calorie intake/high fat diet enhance accumulation of IMCL in skeletal muscle, but characteristics of fat accumulation differ whether it is induced by exercise or by diet. Koh et al. reported that intermyofibrillar lipid droplets were preferentially used over those in subsarcolemma in cross‐country skiers, which implies that lipid droplets between myofibrils are favoured during intense exercise [S4]. Schleh et al. showed that moderate to high‐intensity physical activity increased the number of lipid droplets of myofibrillar region in obese adults [S5]. Although this study included only 36 subjects, it suggests that even in general population, accumulation of lipid droplets in myofibrillar regions might be induced by exercise to meet high energy demand. On the other hand, diet induced IMCL accumulation occurs beneath the sarcolemma, increasing the size of lipid droplets, possibly leading to insulin resistance [S6]. Further exploration is needed on IMCL accumulation depending on types, time, intensity of exercise or specific composition of diet.

Mitochondria are the central modulator of systemic energy metabolism. Mitochondrial biogenesis and degradation via mitophagy determine the mitochondrial contents. Peroxisome proliferator‐activated receptor‐*γ* coactivator 1*α* (PGC‐1*α*), a core transcriptional coactivator involved in mitochondrial biogenesis and oxidation capacity, is predominantly expressed in skeletal muscle and adipose tissue in response to exercise, hunger and cold [41, 42]. Metabolic sensors, such as sirtuin‐1 (SIRT1) and adenosine 5′‐monophosphate‐activated protein kinase (AMPK), are closely related and induce mitochondrial biogenesis through the regulation of PGC‐1*α* activity [42]. Increasing the expression of PGC‐1*α* in muscle delays the onset of muscle atrophy by inhibiting the production of reactive oxygen species (ROS) and reducing chronic inflammation [42, 43]. The deficiency of PGC‐1*α* in adipose tissue resulted in decreased thermogenic and mitochondrial genes and developed insulin resistance in mice fed a high‐fat diet [44].

The enhanced oxidative ability of mitochondria may account for the insulin sensitivity of athletes' skeletal muscle with high contents of IMCL [38]. By contrast, increased fatty acids, either by excessive lipid supply or mitochondrial dysfunction/decreased mitochondrial content within muscle cells, contribute to the elevated production of lipid signalling molecules, resulting in lipotoxicity and insulin resistance [45]. Additionally, an excessive supply of fatty acids might accelerate ROS production by mitochondria and other sources and can damage cells and contribute to insulin resistance in skeletal muscle, despite conflicting results depending on the type, source, production rate and characteristics of the oxidative species [46]. Increased cellular ROS levels also cause mitochondrial dysfunction, promoting a vicious cycle and inducing insulin resistance in skeletal muscle. Moreover, oxidative stress induced by lipid accumulation causes harmful effects, such as the deterioration of redox balance, endoplasmic reticulum (ER) stress, apoptosis and reduced protein synthesis [45].

Both the function and number of mitochondria in muscle may affect the pathogenesis of sarcopenia. Human sarcopenic muscle exhibited reduced expression of genes encoding mitochondrial respiratory complex and decreased enzymatic activity of complex proteins, resulting in impaired oxidative phosphorylation and energy production [47]. Furthermore, human sarcopenic muscle showed reduced expression of genes regulating mitochondrial unfolded protein response (UPRmt), whose function is a repair or salvage of dysfunctional mitochondria [47]. These changes limit the ability of sarcopenic muscles to cope with damages caused by oxidative stress. The accumulation of age‐related mitochondrial DNA mutations may explain mitochondrial dysfunction in sarcopenia and contribute to the upregulation of apoptotic signalling in muscle, leading to muscle loss [48]. In addition, impaired mitochondria in sarcopenic muscle can disrupt calcium homeostasis, inhibiting muscle contraction and triggering cell death in skeletal muscle [49].

The transgenic overexpression of diacylglycerol acyltransferase 1 (DGAT1) in mice, which catalyzes triglyceride synthesis, increased intramyocellular triglyceride but reduced lipid metabolites, such as diacylglycerol (DAG) and ceramide, emulating the athlete's paradox and protecting against high‐fat diet‐induced insulin resistance [50]. In fact, DAG and ceramides, rather than triacylglycerol per se within lipid droplets, are considered to activate stress‐induced serine kinases hindering insulin signalling through insulin receptor substrate 1 (IRS‐1) and the downstream Akt pathway [51]. Further, DAG accumulation contributes to insulin resistance in skeletal muscle by inhibiting the insulin signalling pathway through protein kinase C (PKC) activation; however, isoforms of PKC involved vary depending on the experimental settings [52]. In humans, lipid infusion augmented DAG and PKCθ signalling, thus inhibiting tyrosine phosphorylation of IRS1 [53]. Intracellular ceramides might hamper Akt2 action, probably by activating protein phosphatase 2A (PP2A) that can cause Akt2 dephosphorylation and by hindering the separation of PKCζ from Akt2, thereby inhibiting the activation of Akt2 [54]. Saturated fatty acids, such as palmitate, are known to induce lipotoxicity in muscle, and unsaturated fatty acids may exert a protective effect on muscle atrophy, presumably by increasing insulin sensitivity, decreasing pro‐inflammatory signalling, improving mitochondrial oxidation capacity and producing beneficial eicosanoids [55]. However, further research to investigate the mechanisms of intracellular lipid metabolites on cell signalling in vivo and how structural variations of lipid metabolites affect their actions is needed. Long‐term exercise in athletes may stimulate regular breakdown and resynthesis of the IMTG pool, which results in more efficient coupling of lipolysis to fat oxidation, thereby decreasing the accumulation of toxic lipid metabolites and potentially improving insulin resistance in the skeletal muscle [40].

Toxic effects of intracellular lipid metabolites such as DAG and ceramide have also been reported in cardiac muscle, possibly through impairments in insulin signalling, mitochondrial dysfunction and relevant apoptosis [S7]. Intracellular lipid droplets in cardiomyocytes of rat showed a protective effect against cell death throughout ischemia and reperfusion, which might be due to the dissociation of harmful fatty acids and free Ca^2+^ in cytoplasm within lipid droplets [S8].

Previous studies have demonstrated an association between lipids and sarcopenia. Using Mendelian randomization analysis on human plasma lipid metabolite data, Liu et al. showed that ceramides are a risk factor for low muscle mass and inconsistent results of different types of phosphatidylcholines and phosphatidylethanolamines in muscle; however, the presence of horizontal pleiotropy should be considered regarding the causality between lipid metabolites and sarcopenia [56]. Hsu et al. recently reported metabolic and lipidomic biomarkers of sarcopenia and found that phosphatidylinositol 32:1 had the greatest precision in identifying sarcopenia among older adults [57]. Further studies are needed for validation because the sample size of patients with sarcopenia was small and dietary protein intake and related factors were not assessed. Intracellular lipid accumulation and dyslipidemia are closely linked in the context of insulin resistance [58, 59], and some studies have examined the association between dyslipidemia and sarcopenia. In the Multi‐Ethnic Study of Atherosclerosis (MESA), computed tomography (CT)‐measured muscle mass exhibited an inverse relationship with total cholesterol, triglycerides, very low‐density lipoprotein cholesterol (VLDL‐C) and high‐density lipoprotein cholesterol (HDL‐C) [60]. Bi et al. demonstrated a positive relationship between dyslipidemia and sarcopenia; however, Jiang et al.'s cross‐sectional study reported that lipid profiles, including total cholesterol, triglyceride and low‐density lipoprotein, were significantly lower within normal reference ranges in old adult patients with than those without sarcopenia [61, 62]. Recently, using KNHANES data, we observed that a higher remnant‐C value is associated with an increased risk of low muscle mass in a nation‐wide population‐based study [63].

Fat mass and distribution would be important in metabolic effects, and there are potential gender differences in lipid accumulation and regulation. Higher IMTG concentrations and increased re‐esterification of free fatty acids were observed in women than in men, which implies that women tend to accumulate fatty acids, while men incline to readily utilize them through oxidation [S9, S10]. Men have more muscle mass and are generally more affected by insulin resistance than premenopausal women. This might be explained partially by the differences in fat distribution and effects of sex hormones. Men are more likely to accumulate visceral fat, which is more metabolically unfavourable, whereas women are likely to store fat in subcutaneous area [S11]. In addition, oestrogen may exert protective effects against insulin resistance in premenopausal women [S11]. Holcomb et al. showed greater endurance exercise capacity was observed in female mice compared to male mice, suggesting that female mice are better able to use fatty acids as an energy source during the endurance exercise [S12]. Female muscle tends to be richer in Type 1 fibres and has more mitochondrial contents with associated proteins than male muscle [S13], which may be associated with the increased muscle endurance of female muscle. However, more investigation is needed on sex‐specific differences in muscle composition, effects of sex hormones, their interactions with other hormones and changes in response to specific stimuli.

Intracellular lipid metabolites are important factors in muscle metabolism. Different types of lipid derivatives and alterations in their chemical structure might exert different actions on muscle, requiring further research. Moreover, how intracellular lipids and lipid profiles of dyslipidemia are related in patients with sarcopenia is not fully understood.

## 4 Interplay Between Fat and Muscle: Adipokines and Myokines

**FIGURE 2:**
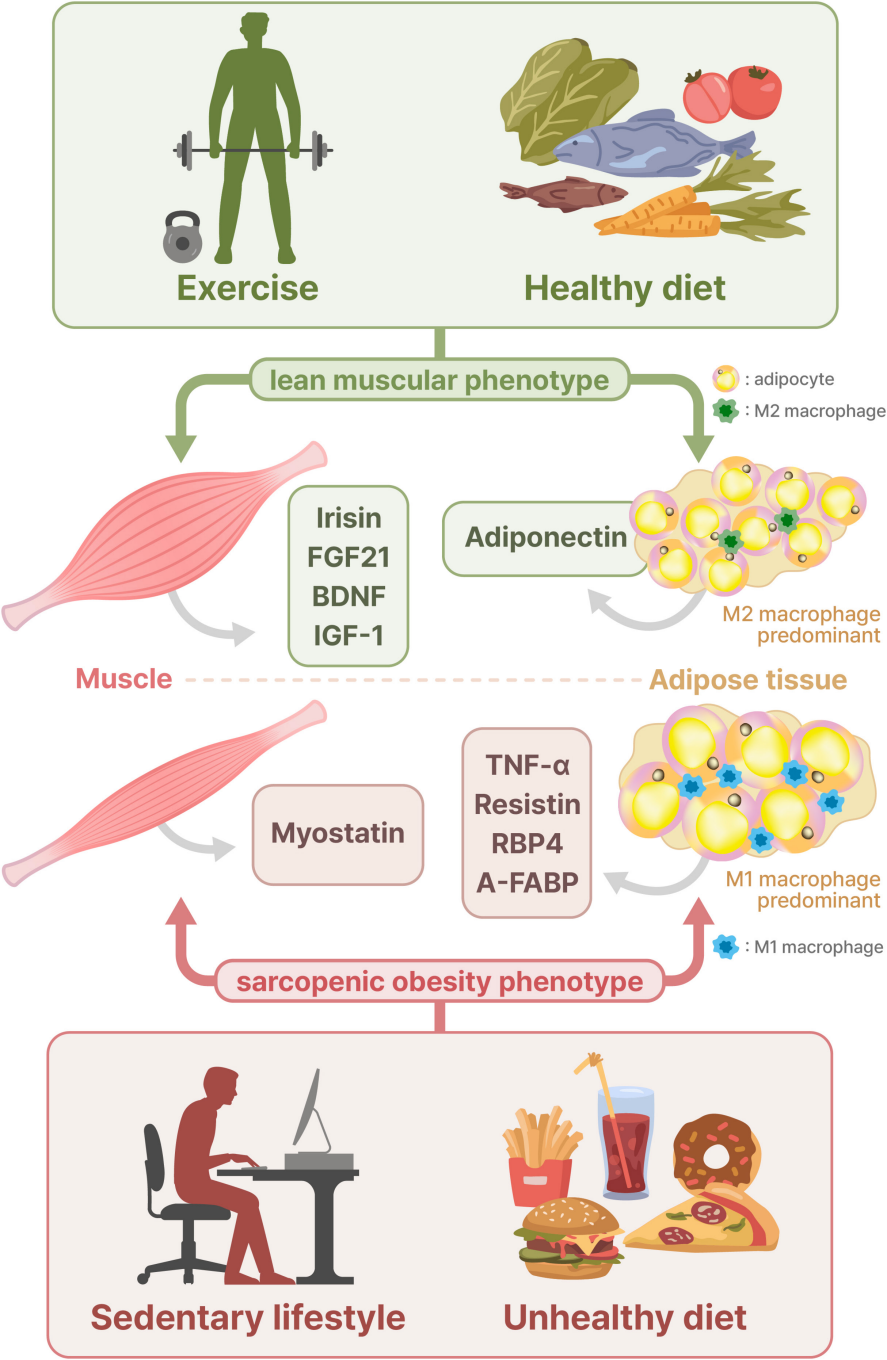
Interconnections between adipose tissue and muscle through adipokines and myokines.

Adipose tissue and skeletal muscle are currently considered endocrine organs that secrete various mediators to communicate with other tissues in the body (Figure 2). Adipokines and myokines are proteins or polypeptides secreted by fat and muscle cells, respectively [17]. The alterations and characteristics of these secreted cytokine profiles may contribute to metabolic abnormalities [64].

Adiponectin is a core adipokine with insulin‐sensitizing, anti‐inflammatory and anti‐atherosclerotic effects, predominantly secreted by adipocytes; however, other cells, including skeletal muscle, also secrete adiponectin [65]. Adiponectin has been assumed to increase intramyocellular calcium influx, thus enhancing muscle contractile force [65]. Additionally, adiponectin has been reported to stimulate AMPK in skeletal muscle, promoting insulin sensitivity [66]. Adiponectin induces glucose uptake and free fatty acid oxidation in skeletal muscle and suppresses gluconeogenesis in the liver [9]. Adiponectin levels could be raised by lifestyle modifications such as diet and exercise, or thiazolidinediones activating PPAR*γ* [67]. Adiponectin receptor 1 (AdipoR1), the predominant receptor isoform in skeletal muscle, is suppressed in obesity and Type 2 diabetes [68]. Exercise ameliorates muscle loss and dysfunction by increasing the circulating adiponectin levels and muscle AdipoR1 expression, accompanied by mitochondrial biogenesis via *the* AMPK/PGC‐1*α* pathway and protein synthesis through the Akt/mTOR pathway [69]. Subclinical inflammation associated with ageing, termed ‘inflammaging’, is implicated in increases in pro‐inflammatory cytokines, such as tumour necrosis factor (TNF)‐*α*, interleukin‐6 (IL‐6), IL‐1*β* and interferon‐*γ*, as well as decreases in anti‐inflammatory cytokines including IL‐10 [70]. Obesity, along with ageing, commonly accompanied by physical inactivity or an unhealthy diet, is associated with low‐grade inflammation through macrophage recruitment and activation, pro‐inflammatory cytokine production and cellular senescence, all of which may contribute to sarcopenia [70]. Moreover, ageing and abdominal obesity increase the ratio of pro‐inflammatory M1 to anti‐inflammatory M2 macrophages [9]. TNF‐*α* suppresses myogenesis by decreasing myogenic regulatory factors, such as myoD and myogenin [71]. Moreover, TNF‐*α* restrains myogenic differentiation in C2C12 cells via the activation of nuclear factor‐*κ*B (NF‐*κ*B) and impairment of the IGF‐1/PI3K/Akt signalling pathway [72]. Further, TNF receptor (TNFR) 1 mediates TNF‐*α* signalling on suppression of AMPK activity through the upregulation of protein phosphatase 2C (PP2C) [73]. This precipitates the suppression of fatty acid oxidation and accumulation of intramuscular diacylglycerol, causing insulin resistance in skeletal muscle [73]. Moreover, PKC, c‐Jun N‐terminal kinase (JNK) and I*κ*B kinase (IKK)/NF‐*κ*B pathways that are stimulated by TNF‐*α* and IL‐1*β* impair insulin signalling in myocytes [74]. Exercise ameliorates inflammation by reducing pro‐inflammatory adipokines, including TNF‐*α*, IL‐6 and monocyte chemoattractant protein‐1 (MCP‐1), and by escalating anti‐inflammatory adiponectin in visceral adipose tissue [75]. Experimental studies have suggested that resistin, a pro‐inflammatory adipokine, appears to inhibit myogenesis via the activation of classical NF‐*κ*B and disturb myogenic differentiation [76, 77]. Additionally, resistin inhibits AMPK activity, contributing to metabolic dysfunction and insulin resistance in rat skeletal muscle cells [78]. Retinol binding protein 4 (RBP4), as an adipokine, has been known to be related to insulin resistance and, probably, induce adipose tissue inflammation [79, 80]. Higher levels of RBP4 are reportedly associated with an increased risk of sarcopenia, and RBP4 negatively correlates with sarcopenic measurements among Chinese older adults, suggesting its role as a potential biomarker of sarcopenia [81]. Zhang et al. recently demonstrated that RBP4 may induce fat infiltration and muscle atrophy by mediating the signalling receptor and transporter of retinol 6 (STRA6) in mice [82]. In their experimental study, RBP4 inhibition exerted a protective effect against fat infiltration and muscle atrophy, implying RBP4 suppression's therapeutic potential in sarcopenia [82]. Adipocyte fatty acid binding protein (A‐FABP) is another adipokine associated with insulin resistance and inflammation [83], which can promote SO. Previous studies have demonstrated that serum A‐FABP levels are positively associated with SO and pre‐sarcopenia in the general population [84, 85].

Myostatin, a representative myokine belonging to the transforming growth factor‐*β* superfamily, is a strong negative modulator of myogenesis, which inhibits the proliferation of myoblasts and activation of satellite cells to induce muscle atrophy [86, 87]. Myostatin suppresses muscle protein synthesis by activating the Smad2 and Smad3 pathway via activin type II receptors (ActRII) and induces muscle atrophy via forkhead box O (FOXO)‐mediated pathway [68]. Furthermore, myostatin may be involved in fat mass regulation. Interestingly, regulatory effects may differ depending on the status of cells; myostatin can suppress the differentiation of preadipocytes into adipocytes, whereas it can foster adipogenesis of mesenchymal stem cells [88]. Myostatin knockout (Mstn −/−) mice demonstrated increased muscle mass and reduced fat mass. Moreover, the browning of white adipose tissue was induced by activating the AMPK‐PGC1*α*‐fibronectin type III domain‐containing protein 5 (FNDC5) pathway in skeletal muscle of mice [89]. Previous studies have demonstrated that higher myostatin levels are observed among people with obesity [90] and the inhibition of myostatin decreases fat mass in rodent models [91], implying its primary effects on fat accumulation. Irisin, which is released through the proteolytic processing of FNDC5, is induced by physical exercise and can promote white adipose tissue browning and thermogenesis mediated by p38 MAPK and extracellular‐regulated protein kinase (ERK) pathway activation [92]. Moreover, irisin increases glucose uptake through the AMPK pathway in skeletal muscle and augments energy expenditure in the adipose tissue, thus inhibiting lipid accumulation and reducing body weight in mice [93]. Irisin promoted myogenic differentiation and myoblast fusion, thus inducing muscle hypertrophy and improving muscle strength in mice [94]. Moreover, in this study, irisin induced the activation of satellite cells and alleviated denervation‐induced muscle atrophy [94]. Fibroblast growth factor 21 (FGF21) induces glucose uptake in adipocytes and stimulates the fat browning of white adipose tissue [95]. FGF21 deficiency upregulated the expression of atrophic factors such as muscle RING finger 1 (MuRF1) and atrogin‐1 and aggravated inflammatory cytokines, accompanied by decreased AMPK phosphorylation in the skeletal muscle of mice fed a high‐fat diet [96]. Interestingly, a multivariate analysis revealed that FGF21 concentration exhibits a negative correlation with muscle strength but no significant association with muscle mass in our multicenter cohort study [97]. Brain‐derived neurotrophic factor (BDNF), secreted following exercise, may promote fat oxidation in skeletal muscle via AMPK activation [98]. In community‐dwelling older adults, circulating BDNF concentrations were significantly associated with frailty after adjusting for confounding factors [99]. Further, BDNF, a regulator of neuromuscular function, has been suggested to be implicated in sarcopenia and SO during the ageing process [9]. We summarized conventional adipokines and myokines with their bidirectional effects in a previous review [S14]. Interaction between fat and muscle is complex as various adipokines and myokines are involved. Novel adipokines and myokines with their actions are being discovered [S15–S24] (Table 1). More studies are needed to uncover their respective effects on the pathophysiology of sarcopenia and their practical clinical implications.

**TABLE 1:** Novel adipokines and myokines with their effects on muscle.

	Effect on muscle	Clinical implication
Adipokine		
Asprosin	Asprosin impairs insulin signalling in skeletal muscle by activating PKC*δ*, which induces ER stress and inflammation [S15].	Increased level of asprosin was observed in those with metabolic diseases such as Type 2 diabetes and obesity, implying its potential as treatment target [S16].
Visfatin	Visfatin upregulates glucose uptake through AMPK‐p38 MAPK pathway in mouse skeletal muscle cells [S17].	Visfatin contributes to insulin sensitivity through insulin‐mimetic action [S18].
Chemerin	Chemerin leads to insulin resistance in skeletal muscle, and this might be related to mitochondrial dysfunction [S19].	Chemerin has pro‐inflammatory properties, and high levels of chemerin were observed in those with metabolic syndrome and obesity [S20].
ANGPTL4	Increased levels of ANGPTL4 mRNA and proteins were observed during myoblast differentiation [12].	ANGPTL4 controls plasma triglyceride levels by suppressing lipoprotein lipase activity [S21].
Myokine		
FNDC1	FNDC1 promotes differentiation of myoblast in vitro and enhances muscle repair in mice [S22].	Administration of recombinant FNDC1 mitigated muscle pathology in a Duchenne muscular dystrophy mouse model, implying its therapeutic potential against muscle diseases.
Decorin	Decorin plays an antagonistic effect to myostatin, thereby leading to muscle growth [S23]. Decorin enhances expression of pro‐myogenic factors such as Myod1 and follistatin [S23].	Decorin may exert anti‐inflammatory effects and is known to prevent angiogenesis/tumorigenesis [S24].

Abbreviations: AMPK, AMP‐activated protein kinase; ER stress, endoplasmic reticulum stress; FNDC1, fibronectin type III domain containing 1; PKC*δ*, protein kinase C‐delta.

Although this review focuses on sarcopenia, it would be intriguing to briefly address cachexia, which shares the important characteristics of muscle wasting and dysregulated metabolism in adipose tissue and skeletal muscle. Primary sarcopenia occurs due to muscle loss and fat accumulation accompanied by ageing, whereas cachexia associated with chronic diseases is characterized by weight loss from muscle and fat loss. In cachexia, anorexia and reduced oral intake promote lipolysis to utilize stored energy within fat. Remodelling from white adipose tissue to brown adipose tissue in cachexia might contribute to increased energy requirements [100], and decreased lipoprotein lipase activity in white adipose tissue accelerates fat atrophy in patients with cachexia [101]. In the process of fat depletion, proteolysis of skeletal muscle may be promoted to meet energy requirements by using amino acids [100]. Pro‐inflammatory cytokines, some of which are released by adipose tissue, may enhance this process, accelerating fat and muscle wasting.

### 4.1 Effects of Weight Management on Sarcopenia Among People With Obesity

Previous studies have demonstrated weight control's impact on sarcopenia among individuals with obesity. Tannir et al. reported that significant weight loss through lifestyle modifications in the obese general population precipitated improvements in the appendicular skeletal mass‐to‐weight ratio and a lower risk of SO [102]. Weight loss by calorie restriction may enhance physical function, probably owing to a reduction in muscular lipid contents and improved mobility because of decreased overall fat mass [103]. However, weight reduction through a hypocaloric diet may result in the loss of not only fat mass but also lean mass, which could be mitigated by incorporating exercise therapy [104]. Adequate protein intake is critical in the process of diet‐induced weight loss, and previous studies have suggested the benefits of high protein intake in preserving muscle mass during weight loss [105]. Nevertheless, further studies are needed to determine the ideal protein intake for calorie restriction in each population based on their characteristics.

Weight control through exercise among individuals with obesity is associated with a reduction in body fat mass and enhancement in muscle mass or quality. Exercise may induce the expression and regulation of myokines, adipokines and microRNAs; suppress inflammatory pathways; increase mitochondrial biogenesis; and improve mitochondrial quality, all of which could contribute to muscle hypertrophy, increased muscle strength and quality [106]. Older adults who perform high‐intensity physical activity exhibited a decreased risk of SO [107] and beneficial effects on the skeletal muscle index and hand grip strength [108] compared to those who perform less vigorous physical activity. While this study used validated data with a large sample size, it was unable to establish causality due to its cross‐sectional design. Although aerobic exercise can decrease body fat mass and exert favourable effects on muscle quality and physical function [109], resistance exercise seems more beneficial with respect to healthy body composition [110]. Moreover, resistance exercise enhanced physical performance among patients with SO [111], and elastic band training may be preferentially recommended among resistance exercise types for fat loss and muscle gain [110].

### 4.2 Effects of Lipid‐Lowering Drugs on Sarcopenia

Statin is a widely used lipid‐lowering drug with significant benefits in decreasing cardiovascular risk. However, approximately 7%–29% of patients receiving statin treatment reported experiencing muscle‐related side effects [112]. The detailed mechanisms of statin‐related myopathy are not well understood; however, previous studies have suggested that mitochondrial dysfunction, probably related to decreased coenzyme Q 10 levels, direct inhibition of the electron transport chain complex and apoptosis promotion may play an important role in mediating the negative effects of statin on muscle [112]. Scott et al. reported that statin users exhibit worse muscle performance deterioration without concomitant muscle loss in community‐dwelling older adults than non‐users over a 2.6‐year mean follow‐up period [113]. Matsumoto et al. demonstrated that statin use is adversely associated with muscle strength recovery but not with the recovery of muscle mass in patients with sarcopenia after stroke [114]. In this study, the outcomes were evaluated at discharge, so the long‐term effects of statin use could not be assessed. Studies with various follow‐up durations are needed to determine the transient or long‐term effects of statins on muscle. Also, further research is needed to determine whether the effects of statins vary depending on age and whether there are differences between age‐related and disease‐related sarcopenia. By contrast, the protective effects of statin on sarcopenia have been reported among patients with chronic kidney disease [115] and heart failure [116]. These studies targeted specific ethnic populations with concomitant diseases and were limited in establishing underlying mechanisms of statins' effects in respect to comorbidities and drug interactions. Notably, statin users exhibited significant lean mass gains after resistance exercise in the older adult population [117].

## 5 Clinical Strategies for Sarcopenia

Currently, there is no approved pharmacological agent specifically targeting sarcopenia. Therefore, exercise and nutritional interventions are the mainstream of sarcopenia management in clinical fields. Resistance training is primarily recommended to patients with sarcopenia due to its effectiveness in improving muscle mass, strength and performance [118]. Because much of the current evidence is based on aged individuals with no or mild sarcopenia, further research is needed to determine whether the effects of resistance exercise vary with the severity of sarcopenia or in different age groups. Combination with other types of exercise such as aerobic/balance/stretching may help improve physical function, but research on respective effects to sarcopenia is limited [119]. Personalized approach based on age, sex, underlying diseases and general health condition is needed when prescribing exercise to patients with sarcopenia, but there are no specific universal guidelines. Intake of adequate calorie and protein would be recommended to older adults with sarcopenia. Supplementation with certain amino acids, vitamin D and omega‐3 may be beneficial, although the doses and administration methods are not standardized and evidence base is relatively weak [119]. The integration of physical exercise and nutritional support is expected to have an additional effect in improving sarcopenia compared to either intervention alone, but further research is needed to determine the differences in synergistic effects according to the combination of various types of exercise and diet. Moreover, clinicians should consider anabolic resistance and the tendency for low compliance when implementing sarcopenia managements in older adults.

## 6 Conclusion and Perspectives

In this review, we elucidated how fatty infiltration between and within muscle influences skeletal muscle structurally and metabolically. The distribution, location and structure of muscular fatty infiltration may affect their actions on muscle; however, currently, measuring these accurately is challenging. More research is still needed to understand how different aspects of muscle fat accumulation affect skeletal muscle and how their actions vary based on individual medical characteristics. Additionally, we illustrated how lipids function within muscle cells with detailed molecular mechanisms, especially in the context of sarcopenia development. Lipid metabolism is a dynamic process to maintain energy homeostasis and is complicated, involving various regulatory proteins. Moreover, the variety of lipid metabolites makes it more complex to determine the main contributors to muscle health. Further mechanistic studies are needed to elucidate how each lipid metabolite works and interacts in the active metabolism of muscle cells. Additionally, adipose tissue and muscle are considered endocrine organs, and many adipokines and myokines are being discovered; however, their actions, interactions and regulatory processes remain significantly underexplored. Improvements in lipidomics, proteomics technology and imaging modalities may help investigate the specific mechanisms whereby adipose tissue and lipid metabolites affect skeletal muscle.

To prevent and manage sarcopenia, the current main approaches are exercise and nutrition, but optimal recommendation guidelines for lifestyle modifications based on individual medical backgrounds are lacking. Additional interventional studies with larger numbers of participants, various medical conditions and longer follow‐up durations are needed. Further mechanistic studies are necessary to evaluate the effects of statins or other lipid‐lowering agents on muscle composition, quality and function to elucidate the observational studies' conflicting results.

## Ethics Statement

The authors have nothing to report.

## Conflicts of Interest

The authors declare no conflicts of interest.

## Supporting information


**Data S1** Supplementary Information.
