# Supplementary material for: Impact of Adipose Tissue and Lipids on Skeletal Muscle in Sarcopenia
Source: J Cachexia Sarcopenia Muscle. 2025 Jul 10;16(4):e70000. doi: 10.1002/jcsm.70000 (PMC12246390; doi:10.1002/jcsm.70000)
Supplement: Supplementary file 1 — Data S1 Supplementary Information. [file JCSM-16-e70000-s001.docx]

**Supplementary references**

S1. Thanassoulis, G., et al., *Pericardial fat is associated with prevalent atrial fibrillation: the Framingham Heart Study.* Circ Arrhythm Electrophysiol, 2010. **3**(4): p. 345-50.

S2. Kenchaiah, S., et al., *Pericardial Fat and the Risk of Heart Failure.* J Am Coll Cardiol, 2021. **77**(21): p. 2638-2652.

S3. Kashiwagi-Takayama, R., et al., *Myocardial fat accumulation is associated with cardiac dysfunction in patients with type 2 diabetes, especially in elderly or female patients: a retrospective observational study.* Cardiovasc Diabetol, 2023. **22**(1): p. 48.

S4. Koh, H.E., et al., *Pronounced limb and fibre type differences in subcellular lipid droplet content and distribution in elite skiers before and after exhaustive exercise.* J Physiol, 2017. **595**(17): p. 5781-5795.

S5. Schleh, M.W., et al., *Both moderate- and high-intensity exercise training increase intramyocellular lipid droplet abundance and modify myocellular distribution in adults with obesity.* Am J Physiol Endocrinol Metab, 2023. **325**(5): p. E466-E479.

S6. Andrich, D.E., et al., *Altered Lipid Metabolism Impairs Skeletal Muscle Force in Young Rats Submitted to a Short-Term High-Fat Diet.* Front Physiol, 2018. **9**: p. 1327.

S7. Schulze, P.C., K. Drosatos, and I.J. Goldberg, *Lipid Use and Misuse by the Heart.* Circ Res, 2016. **118**(11): p. 1736-51.

S8. Barba, I., et al., *Effect of intracellular lipid droplets on cytosolic Ca2+ and cell death during ischaemia-reperfusion injury in cardiomyocytes.* J Physiol, 2009. **587**(Pt 6): p. 1331-41.

S9. Broussard, J.L., et al., *Sex Differences in Insulin Sensitivity are Related to Muscle Tissue Acylcarnitine But Not Subcellular Lipid Distribution.* Obesity (Silver Spring), 2021. **29**(3): p. 550-561.

S10. Varlamov, O., C.L. Bethea, and C.T. Roberts, Jr., *Sex-specific differences in lipid and glucose metabolism.* Front Endocrinol (Lausanne), 2014. **5**: p. 241.

S11. Ciarambino, T., et al., *Gender Differences in Insulin Resistance: New Knowledge and Perspectives.* Curr Issues Mol Biol, 2023. **45**(10): p. 7845-7861.

S12. Holcomb, L.E., et al., *Sex differences in endurance exercise capacity and skeletal muscle lipid metabolism in mice.* Physiol Rep, 2022. **10**(3): p. e15174.

S13. Rosa-Caldwell, M.E. and N.P. Greene, *Muscle metabolism and atrophy: let's talk about sex.* Biol Sex Differ, 2019. **10**(1): p. 43.

S14. Park, M.J. and K.M. Choi, *Interplay of skeletal muscle and adipose tissue: sarcopenic obesity.* Metabolism, 2023. **144**: p. 155577.

S15. Jung, T.W., et al., *Asprosin attenuates insulin signaling pathway through PKCdelta-activated ER stress and inflammation in skeletal muscle.* J Cell Physiol, 2019. **234**(11): p. 20888-20899.

S16. Farrag, M., et al., *Asprosin in health and disease, a new glucose sensor with central and peripheral metabolic effects.* Front Endocrinol (Lausanne), 2022. **13**: p. 1101091.

S17. Lee, J.O., et al., *Visfatin, a novel adipokine, stimulates glucose uptake through the Ca2 +-dependent AMPK-p38 MAPK pathway in C2C12 skeletal muscle cells.* J Mol Endocrinol, 2015. **54**(3): p. 251-62.

S18. Abdalla, M.M.I., *Role of visfatin in obesity-induced insulin resistance.* World J Clin Cases, 2022. **10**(30): p. 10840-10851.

S19. Xie, Q., et al., *Chemerin-induced mitochondrial dysfunction in skeletal muscle.* J Cell Mol Med, 2015. **19**(5): p. 986-95.

S20. Helfer, G. and Q.F. Wu, *Chemerin: a multifaceted adipokine involved in metabolic disorders.* J Endocrinol, 2018. **238**(2): p. R79-R94.

S21. Sukonina, V., et al., *Angiopoietin-like protein 4 converts lipoprotein lipase to inactive monomers and modulates lipase activity in adipose tissue.* Proc Natl Acad Sci U S A, 2006. **103**(46): p. 17450-5.

S22. Zhang, R.X., et al., *FNDC1 is a myokine that promotes myogenesis and muscle regeneration.* EMBO J, 2025. **44**(1): p. 30-53.

S23. Lee, J.H. and H.S. Jun, *Role of Myokines in Regulating Skeletal Muscle Mass and Function.* Front Physiol, 2019. **10**: p. 42.

S24. Akbulut, M.C. and O. Erbaş, *Possible therapeutics: Myokines.* Demiroglu Science University Florence Nightingale Journal of Transplantation, 2022. **7**(1): p. 032-039.
